# P-1891. Outcomes of a Learning Needs Assessment Survey in the Development of the Extension for Community Health Outcomes Model for HIV Training in Midwestern Non-Metropolitan Areas

**DOI:** 10.1093/ofid/ofaf695.2060

**Published:** 2026-01-11

**Authors:** Duncan Works, Dan Cramer, Emmanuel Nazaire Essam Nkodo, Elizabeth Lyden, Renae Furl, Yue Zhan, Jennifer O’Neil, Maureen Kubat, Heather Saarela, Nada Fadul, Jennifer M Davis

**Affiliations:** University of Nebraska - Medical Center, Lincoln, NE; University of Nebraska - Medical Center, Lincoln, NE; University of Nebraska Medical Center, Omaha, NE; University of Nebraska Medical Center, Omaha, NE; University of Nebraska - Medical Center, Lincoln, NE; University of Nebraska - Medical Center, Lincoln, NE; University of Nebraska - Medical Center, Lincoln, NE; University of Nebraska Medical Center, Omaha, NE; University of Nebraska - Medical Center, Lincoln, NE; University of Nebraska Medical Center, Omaha, NE; University of Nebraska Medical Center, Omaha, NE

## Abstract

**Background:**

Of the approximately 2,500 people living with HIV (PWH) in Nebraska in 2020, 23% resided in rural areas with rural HIV diagnoses nearly doubling in 2021, accounting for 35% of new cases. PWH in rural areas have delayed diagnosis and poor access to care. In response, the University of Nebraska Medical Center Specialty Care Clinic implemented an Extension for Community Healthcare Outcomes (ECHO) model to enhance rural provider education and support through virtual lectures and case-based learning.

**Figure:**
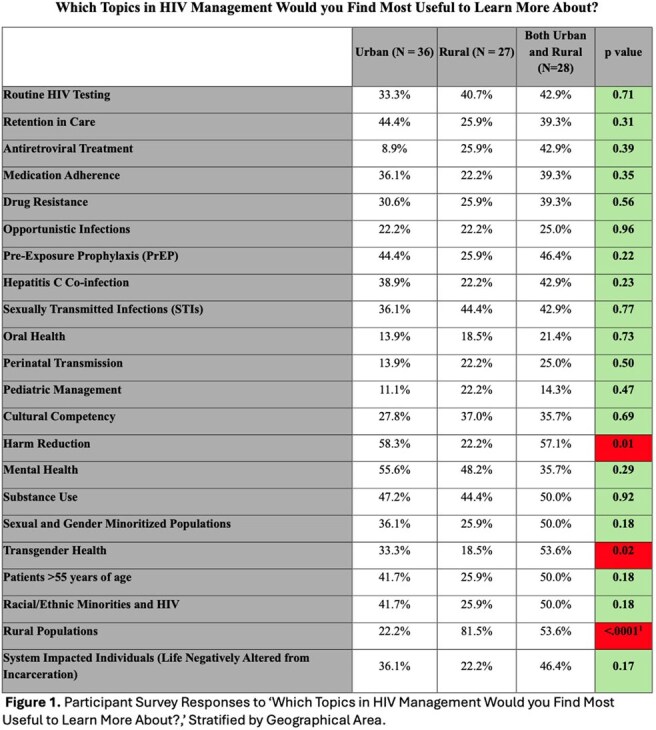

**Figure:**
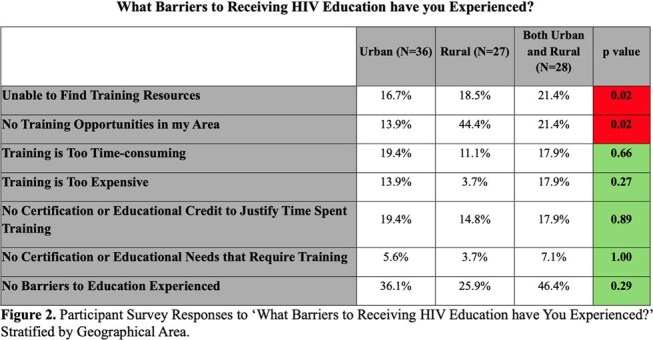

**Methods:**

An anonymous, cross-sectional survey was distributed to healthcare professionals engaged in HIV care in Nebraska. Survey domains included demographics, clinical role and setting, years of experience, geographic region, perceived barriers to HIV education, and topic preferences. Associations were assessed using Fisher’s exact or Chi-square tests (p< 0.05), with analyses conducted in SAS 9.4.

**Figure:**
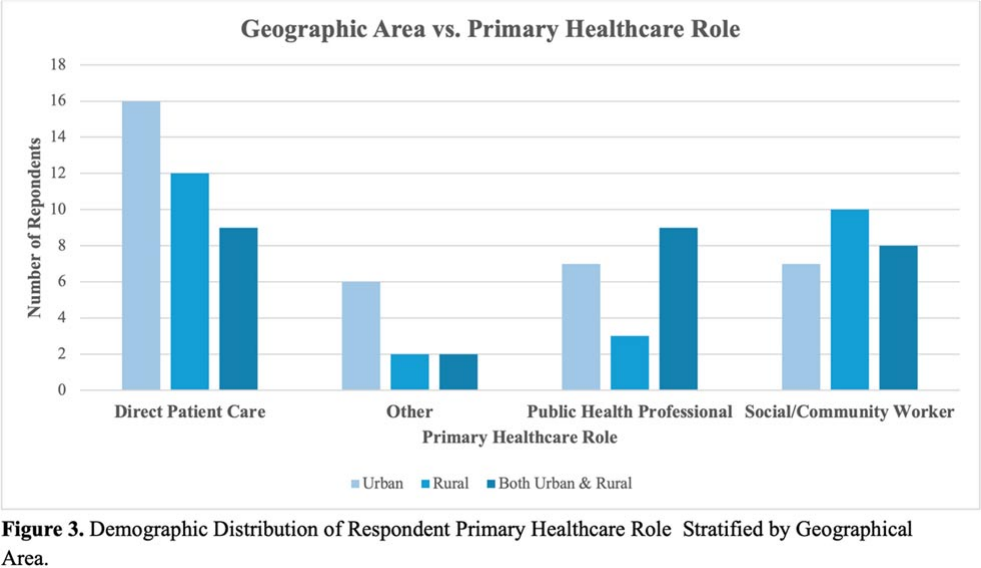

**Figure:**
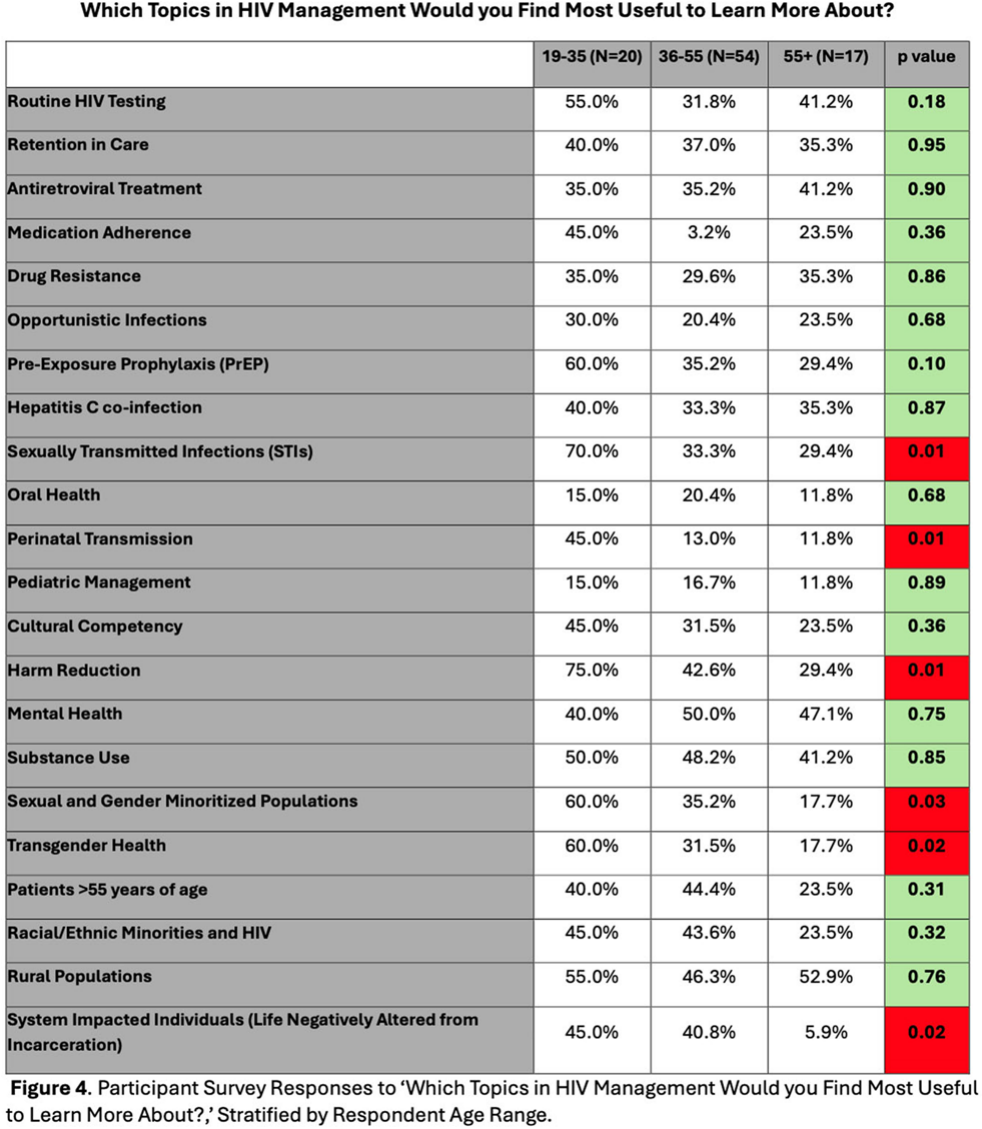

**Results:**

Of the 91 survey respondents, 59.3% were aged 35-55, with 22.0% aged 19–34 and 19.7% over 55. Healthcare roles included 40.7% direct patient care, 27.5% social/community health workers, 20.9% public health professionals. About a third (39.6%) served in urban areas, 29.7% rural, 30.8% both. Rural respondents reported greater barriers to accessing HIV education (p< 0.02). Urban professionals expressed higher interest in Harm Reduction (p< 0.008) and Transgender Health (p< 0.02), while rural respondents prioritized Rural Health topics (p< 0.0001). Professionals aged 19–35 more frequently identified educational needs in Perinatal Transmission (p< 0.006), Transgender Health (p< 0.02), Sexual and Gender Minoritized Populations (p< 0.03), Harm Reduction (p< 0.01), and System-Impacted Individuals (p< 0.02). Public Health and Social/Community Workers reported higher interest in topics of Retention in Care (p< 0.03), Sexual and Gender Minority Populations (p< 0.0002), and Transgender Health (p< 0.03).

**Conclusion:**

Rural providers faced greater barriers to accessing HIV training, indicating an urgent need to expand educational resources in underserved areas. Our findings highlight the need for diverse and adaptable HIV educational initiatives tailored to professional, geographic, and generational differences and will inform future educational interventions.

**Disclosures:**

Dan Cramer, APRN-NP, Viiv Healthcare: Grant/Research Support Renae Furl, MPH, Viiv Healthcare: Grant/Research Support Jennifer O'Neil, RN, BSN, Viiv Healthcare: Grant/Research Support Heather Saarela, BSPH, Viiv Healthcare: Grant/Research Support Nada Fadul, MD, ViiV Healthcare: Advisor/Consultant|ViiV Healthcare: Grant/Research Support Jennifer M. Davis, MD, Viiv: Grant/Research Support

